# Timing of First Exposure to Maternal Depression and Adolescent Emotional Disorder in a National Canadian Cohort

**DOI:** 10.1371/journal.pone.0033422

**Published:** 2012-03-26

**Authors:** Kiyuri Naicker, Maeve Wickham, Ian Colman

**Affiliations:** 1 School of Public Health, University of Alberta, Edmonton, Alberta, Canada; 2 Department of Epidemiology and Community Medicine, University of Ottawa, Ottawa, Ontario, Canada; Federal University of Rio de Janeiro, Brazil

## Abstract

**Objective:**

Correlations have been reported between behavioral and cognitive outcomes in adolescence and exposure to maternal depression during the first postpartum year, but the effects of timing of maternal depression during subsequent exposure periods have rarely been controlled for. This study aims to methodically investigate the importance of timing of initial exposure to maternal depression with respect to adolescent mental health outcomes.

**Methods:**

This study used data on 937 children from the National Longitudinal Study of Children and Youth (NLSCY), a nationally-representative longitudinal survey established in 1994 by Statistics Canada. Ordinal logistic regression was used to confirm associations between adolescent emotional disorder (at 12–13 years) and initial exposure to maternal depression during 2-year intervals from birth to adolescence. Following their initial exposure to maternal depression, children were dropped from subsequent cycles. Stressful life events, chronic health conditions, maternal alcohol use, maternal marital status, gender, and SES were included as covariates.

**Results:**

The results indicated that adolescents who were initially exposed to maternal depression between the ages of 2–3 years and 4–5 years had a two-fold increase in odds of emotional disorder. No increase in odds was observed in those initially exposed during the first postpartum year or later in childhood.

**Conclusions:**

The results demonstrate that a sensitive period of initial exposure to maternal depression may occur between the ages of 2 and 5, and not during the first year of life indicated by previous research. These findings are congruent with the literature on emotional and behavioral development in early childhood.

## Introduction

Maternal depression has been identified as an important risk factor for childhood psychopathology [1], [2], with numerous studies reporting a two- to three-fold increase in risk of emotional disorders in children of affected parents [3], [4]. These effects persist across the life course, as offspring of depressed parents are at least three times more likely to develop anxiety disorders, major depression, and substance dependence 20 years later [2]. A substantive body of literature points to the diverse causal mechanisms underlying this association, which can be grouped under the main themes of genetic vulnerability, *in utero* effects on neuroregulation, adverse parenting behaviours, and stressful environmental contexts [5]. Mechanisms sorted into the former two themes exert themselves prenatally, and those associated with the latter two come into play primarily during the postnatal period. However, despite the focus on these rather broad epochs of early development, a detailed examination of the impact of maternal depression during distinct periods in early life and throughout childhood is still lacking. As such, little progress has been made in arriving at a clear developmental model for the intergenerational transmission of depression risk.

The first year of life is frequently examined as a “sensitive” period for exposure to maternal depression, and a preponderance of research has focused on the various effects of postpartum depression on the developing infant [6]–[9]. To a lesser extent, exposure to maternal depression during childhood has also been explored, though primarily during the preschool years [10]–[13]; studies examining exposure in later childhood are rare [14]–[16]. In longitudinal studies, the outcomes of postpartum depression exposure have been ascertained at a wide range of ages, spanning from 3 months to 15 years [14], [17]–[19], and have demonstrated compelling effects of postpartum depression on the neurophysiological, cognitive, behavioural and affective development of the offspring [20]–[22].

Significantly, the postpartum period coincides with the major period of infantile bonding described by attachment theorists [23], [24]. During this time, infants will transition from indiscriminate and instinctual signaling to focus their interactions increasingly on primary caregivers [25]. Through parental responsiveness and continued engagement in social interaction, these exchanges will culminate in the formation of an attachment bond and the establishment of one of the first relationships in early life. Researchers have expressed a keen interest in the effects of postpartum depression on the developing attachment bond; a number of studies have demonstrated that the quality of attachment in infants of depressed parents is often compromised [26], [27]. As a result, these offspring are at a significantly higher risk of developing insecure or avoidant attachment styles, the consequences of which are thought to include internalizing disorders, conduct disorders, anxiety, and a range of antisocial behaviours [28]–[30].

However, despite the wealth of evidence which demonstrates long-term effects of postpartum depression, a lack of identification and treatment of distinct and varied exposure periods to maternal depression exists [5]. It is therefore possible that this association may actually be explained by lingering symptoms that persist into important development periods during early childhood. Notably, few long-term mental health studies have attempted to examine adolescents who systematically vary in their age of first exposure to maternal depression. Studies such as these are necessary to determine precisely when maternal depression will exert its most pernicious effects, and under what conditions the impact will extend beyond the childhood years.

The purpose of this paper was to investigate whether the effects of maternal depression on adolescent mental health (as indicated by the presence of an adolescent emotional disorder) are dependent on the period of childhood development during which initial exposure to maternal depression occurs. We hypothesized that 1) sensitive periods to maternal depression would be apparent during early childhood and 2) that the effect of exposure during the postpartum period would be less pronounced after accounting for subsequent exposure periods.

## Methods

### Ethics statement

This study was approved by the Health Research Ethics Board of the University of Alberta. Written informed consent was obtained from parents of survey members by Statistics Canada (see below).

### Study Sample

This study used data from the National Longitudinal Survey of Children and Youth (NLSCY), a longitudinal survey established by Statistics Canada in 1994/95 in order to investigate child health and development [31]. Participants were selected using a stratified, multistage probability design which utilized Canadian residences as sampling units, and which yielded a representative initial cohort of 16,903 children [32]. For estimation purposes, sampling weights were devised for each child based on the inverse of his or her probability of selection and enlistment into the study [33]. The NLSCY is a rich source of data on health, behavior, physical development and demographic and social factors, augmented every two years by follow-up data collection cycles.

The current study included 937 participants who: joined the survey in their first year of life; completed a self-report depression and anxiety questionnaire at age 12 or 13; and whose parental respondent was their biological mother. In addition to facilitating comparisons with previous studies, the latter requirement was considered especially important as a majority of caregivers are women, and women are almost twice as likely to develop unipolar depression as men [34]. Data on each child was obtained from the initial survey cycle through to cycle 7 (in 2006/07). Additional details on the design and methodology of the NLSCY are available online at: http://www.statcan.gc.ca/cgibin/imdb/p2SV.pl?Function=getSurvey&SDDS=4450

### Adolescent Emotional Disorder

The NLSCY includes an emotional disorder-anxiety scale for children and youth, based on seven items taken from the Ontario Child Health Study [32]. This scale was derived from symptom criteria for the DSM-III-R [35]. In youth ages 12 and over, this scale assesses self-reported symptoms of anxiety and depression [36] using the following statements: “I am unhappy or sad”, “I am not as happy as other people my age”, “I am too fearful or nervous”, “I worry a lot”, “I cry a lot”, “I am nervous, high-strung or tense”, and “I have trouble enjoying myself.” Each statement included three response categories - “never or not true”; “sometimes or somewhat true”; or “often or very true.” Each response was assigned a score of 0 to 2 (high scores indicating a greater degree of emotional disorder). Of a total possible score of 14, subjects were divided into four score groups– these centiles corresponded to severity of symptoms experienced (0–50%, no symptoms; 51–75%, mild symptoms; 76–90%, moderate symptoms; and 91–100%, severe symptoms) [37].

### Maternal Depression

An abbreviated version of Radloff's Centre for Epidemiological Studies Depression Scale (CES-D-12) [38] was administered every two years to the parent or person most knowledge (PMK) about the child. Each item relates to a particular feeling or behavior over the previous week, and has four response categories: “rarely or none of the time,” “some or a little of the time (1 or 2 days),” “occasionally or a moderate amount of time (3 or 4 days),” or “most or all of the time (5 to 7 days).” Each item was assigned a score of 0 to 3 (scoring was reversed for three questions which had negative loading), yielding a total possible score of 36. In order to produce a cut-off proportional to that of the 20-item CES-D (where scores range from 0 to 60 and a score of 16 represents a classification of depression), the 12-item score was dichotomized based on the method described by Somers and Willms [39]. Mothers scoring above a cut-off of 9 were classified as depressed, and those falling below this value classified as non-depressed. The Cronbach's alpha of the 12-item scale is 0.82, only slightly lower than the reliability of the full 20-item scale (0.85) [32].

“Initial” exposure to maternal depression was defined as the age at which the first incidence of maternal depression occurred during the child's life. Following this initial exposure, children were dropped from remaining study cycles.

### Covariates

Covariates were selected *a priori* due to their associations with adolescent depression. Mothers reported on 15 stressful life events the child may have experienced in the preceeding 2 years (including death of a family member, divorce/separation of parents, illness/injury of child, abuse/fear of abuse, and others). Heavy maternal alcohol use was indicated by the consumption of either >10 drinks a week, or binge drinking at a frequency higher than once per month (i.e., 5 or more drinks on one occaison) [40], [41]. Child chronic health problems [42] were reported by the mother (including diabetes, heart disease, and epilepsy). Maternal chronic health conditions [43] and marital status [44] were self-reported. Concurrent maternal depression was also included as a covariate, to ensure that the observed associations with initial periods of exposure would not be masked by the proximal effects of maternal depression. All analyses were also adjusted for gender [45] and socio-economic status as measured by household income adequacy (a two-level variable derived by Statistics Canada) [32]. All covariates were assessed in Cycle 7 (2006/07) when subjects were 12–13 years old.

### Data analysis

Ordinal logistic regression was used to confirm associations between emotional disorder in adolescence (12–13 yrs) and initial exposure to maternal depression at each cycle. Odds ratios were reported to determine whether, and to what magnitude, initial exposure at each stage was associated with adolescent emotional disorder. Multivariate ordinal logistic regression was used to account for the effect of covariates on this association. In each case, the proportional odds assumption was tested and held. Hosmer-Lemeshow goodness-of-fit tests were performed on the final regression models (p>0.05). The correlation coefficients for all predictor variables were inspected using a correlation matrix, and were determined to be sufficiently low that issues of multicollinearity were not indicated (the highest correlation between predictor variables was below 0.35). As per the Statistics Canada data publication guides, our analyses included the recommended population weights [33]. All analyses were performed using STATA 10.

## Results

The original cohort for this study had a 12-year follow-up rate of 65%, yielding a study sample of 937 adolescents. Of these, 49.5% were female and the majority came from middle or high income households (90.4%) (Table 1). 37.8% had experienced a stressful life event in the previous two years and 31.2% had a reported chronic health condition (Table 1). 38.2% had mothers who were depressed at least once during their lifetime; of these, 14.8% were depressed when their children were 12–13 years old (Table 1). An assessment of maternal characteristics revealed that 30.6% were unmarried, just under half reported some form of chronic health condition (43.5%), and 6.2% reported heavy alcohol use within the previous year (Table 1).

**Table 1:** Odds Ratios for Adolescent Emotional Disorder Associated with Demographic and Health Factors in Participants Aged 12–13 Years (Bivariate Analysis).

Risk Factor	Prevalence (%) or Mean (N = 937)	Emotional Disorder at Age 12–13 (OR [95% CI]) (Weighted)	
		Crude	Adjusted†
***Adolescent Characteristics***			
Gender (Female)‡	49.5%	**1.76 [1.21–2.55]** **	**1.76 [1.21–2.55]** **
Low Household SES‡	9.6%	1.46 [0.55–3.92]	1.46 [0.55–3.92]
Chronic Health Condition	31.8%	1.17 [0.78–1.76]	1.29 [0.86–1.92]
Stressful Life Events (last 2 years)	37.8%	1.43 [0.98–2.07]	**1.48 [1.02–2.15]** **
***Maternal Characteristics***			
Marital Status (Unmarried)	30.6%	1.31 [0.88–1.96]	1.39 [0.90–2.15]
Chronic Health Condition	43.5%	1.26 [0.84–1.88]	1.23 [0.82–1.83]
Alcohol Use (Heavy)	6.2%	1.45 [0.88–2.40]	1.70 [1.00–2.89]
Social Support (below mean)	19.7	0.95 [0.89–1.01]	0.95 [0.90–1.01]
≥1 Depressive Episode	38.2%	**1.80 [1.24–2.59]** **	**1.83 [1.27–2.63]** **
Currently Depressed	14.8%	**2.02 [1.17–4.16]** *	**2.17 [1.17–4.03]** *

† Adjusted for gender and SES.

‡ Gender - adjusted for SES only; SES - adjusted for gender only.

* Statistically significant at p<0.05 level.

** Statistically significant at p<0.01 level.

The adjusted odds ratio for experiencing an emotional disorder during adolescence, following exposure to maternal depression at any point, was 1.83 (95% CI, 1.27–2.63). These odds were even higher in those subject to current exposure (OR = 2.17; 95% CI, 1.17–4.03) (Table 1). Girls reported significantly higher odds of suffering from emotional disorder than did boys during adolescence (OR = 1.76; 95% CI, 1.21–2.55) (Table 1). No significant increase in odds was observed in adolescents according to socioeconomic status, mother's marital status, maternal alcohol abuse, or the presence of a chronic health condition in either the mother or child (Table 1). However, adolescents who had experienced a stressful life event within the preceding two years had 1.48 times the odds of suffering from emotional disorder (95% CI, 1.02–2.15) (Table 1).

Examination of initial exposure to maternal depression by study cycle revealed an almost two-fold increase in odds of emotional disorder in adolescents initially exposed at age 2–3 (OR = 1.87; 95% CI, 1.05–3.53) or 4–5 years (OR = 1.89; 95% CI, 1.14–3.13), after controlling for current maternal depression (Table 2). Initial exposure during any other cycle, including the post-partum period, was not significantly associated with emotional disorder at age 12–13 years (Table 2).

**Table 2:** Association between Adolescent Emotional Disorder and Initial Exposure to Maternal Depression, by Two-Year Intervals.

Age at Initial Maternal Depression Exposure	Prevalence of Maternal Depression by Child Age Group(N = 937)	Emotional Disorder at Age 12–13 (OR [95% CI])		
		Crude	Adjusted for SES and Gender	Multivariate Regression†
<1 year	19.3%	1.64 [0.95–2.83]	1.57 [0.95–2.59]	1.36 [0.80–2.32]
2–3 years	17.3%	**1.98 [1.05–3.76]** *	**2.13 [1.16–3.89]** **	**1.87 [1.05–3.53]** *
4–5 years	15.3%	**2.00 [1.16–3.46]** *	**2.05 [1.21–3.47]** **	**1.89 [1.14–3.13]** **
6–7 years	13.2%	1.46 [0.75–2.81]	1.45 [0.75–2.79]	1.24 [0.60–2.56]
8–9 years	13.3%	1.43 [0.75–2.75]	1.51 [0.82–2.80]	1.19 [0.65–2.19]
10–11years	12.1%	1.77 [0.99–3.18]	1.65 [0.91–2.99]	1.39 [0.73–2.62]
12–13 years	10.8%	**1.96 [1.06–3.64]** *	**1.97 [1.04–3.73]** *	**N/A**

† Adjusted for SES, Gender, Current Maternal Depression (12–13 years), and Stressful Life Events.

* Statistically significant at p<0.05 level.

** Statistically significant at p<0.01 level.

## Discussion

The results from this prospective study of Canadian children demonstrate that a sensitive period of initial exposure to maternal depression on adolescent emotional disorder appears to exist, and occurs between the ages of 2 and 5. Children initially exposed to maternal depression during this period were almost twice as likely to experience symptoms of depression and anxiety in adolescence as their unexposed counterparts. This is novel in light of other findings in the area, many of which strongly implicate postpartum exposure in the development of affective and behavioural problems in childhood.

There may be methodological reasons for this apparent inconsistency. The first relates to exposure measurement. While the NLSCY rigorously obtains data every 2 years, many previous studies have relied upon a single maternal depression measurement or on erratically spaced measures ranging from months to years apart [17], [46]. This approach may result in inclusion of multiple developmental periods, thereby confounding the apparent effects. A lack of control over the potentially sensitive pre-school period may explain the widely observed heterogeneity in results regarding the effects of postpartum depression on subsequent child development and behavior. This is underscored by the finding that, when antecedent and subsequent episodes of depression are taken into account, postpartum depression has not been found to predict adolescent psychopathology [8]. It may be reasoned that the strength of the effect of postpartum depression weakens as infants get older, when considering the significance of this association in relation to child but not adolescent outcomes [6].

One other study has measured initial maternal depression exposure during the early childhood period [12]. It found that initial exposure during the ages of 2 to 4½ years increased the risk of externalizing but not internalizing symptoms, and then only in girls; however, the authors recommended caution in interpreting these results due to small cell sizes. This study did not follow children beyond the kindergarten age, perhaps missing significant associations with longer-term outcomes. Indeed, research has shown strong links between externalizing problems in early childhood and later adolescent depression and anxiety [47]; in this manner, their findings may be congruent with those illustrated here.

A second methodological explanation for the differences between our results and previous research relates to our specific focus on *initial* exposure to maternal depression. This was a necessary criterion in order to isolate the effects resulting from each exposure period. Although our sensitive period occurs immediately following the generally accepted stage of infantile attachment (6 months to 2 years) [48], children in the 2–5 year age group had experienced no prior maternal distress and were ostensibly able to form healthy attachment bonds during this stage. The experience of separation anxiety or grief is considered to be a normal adaptive response following the loss of an attachment bond, and during an episode of maternal depression infants may experience a loss akin the actual loss of a parent [49]. It may be that the adaptive response is heightened following the establishment of such a bond, rather than during the attachment period itself. A similar response has been witnessed in adults; the avoidance of attachment is associated with decreased symptoms of grief in bereaved individuals [50]. It is of note that our findings are in keeping with the tenets of modern attachment theory, and the importance of early experience is still very much stressed by these results.

There are a host of developmental reasons for the long-term effects of exposure to maternal depression during this period. For toddlers and preschoolers, parents are instrumental in the formation of an accurate understanding of social and emotional situations [51]. Importantly, as the child progresses to the toddler years, their cognitive development begins to progress rapidly, expressed through symbolic thought, words, and play [52]. At this stage, the mother is able to act as a guide in extending these cognitive skills through the use of symbolic play and shared reading activities [53], [54]. There is an established link between diminished cognitive ability and depression throughout life [55], and children with lower IQs are significantly more likely to experience later depression and anxiety [56], [57]. It is possible that, at this age of increased cognitive growth and capacity, children may be more sensitive to the decreased engagement associated with maternal depression than they are during early infancy.

Fortunately, some of the effects of maternal depression may be minimized. As noted by Sroufe et. al [58], adaptation is always the joint product of current circumstances and early history; quality of infant care is therefore an important influencer of future pathology, and may not be limited entirely to the confines of parental care [59]. Developmentally oriented early-intervention programs for depressed mothers and infants have been trialed in the United States [60]–[62] and the Netherlands [63], and have been found to help considerably in infant outcomes. Programs such as these are encouraged to continue focusing their efforts on preventing exposure to maternal depression in early childhood, particularly in the vulnerable 2–5 year age group.

Several limitations of this study must be noted. As with many longitudinal studies, attrition was observed; however, additional analysis confirmed that those who dropped out did not differ significantly from the remaining group in exposure to postpartum depression, SES, gender or any other maternal variables (data not shown). The CES-D is not a clinical measure of maternal depression, but a valid screening tool often employed in population-based research. Future research which includes a confirmatory diagnostic interview for maternal depression is therefore needed to strengthen these findings. Likewise, the NLSCY does not provide a clinical measure of adolescent depression but rather provides a measurement of symptoms on a continuous scale. This may affect the generalizability of our results; however, it was also advantageous, as we were able to include children who experienced moderate and/or sub-threshold symptoms in our analysis. Sub-threshold symptoms are associated with future severe depression, as well as functional or psychosocial impairment [64], [65]. In addition, paternal characteristics were not considered here and their inclusion would add depth to future studies.

These limitations were offset by several considerable strengths. This study utilized prospectively collected data on a large, population-based sample of Canadian children. The potential for recall bias was effectively minimized, and exposure measurement occurred at regular 2-year intervals. All analyses were based on high quality data, which were available for a range of major related risk factors. The 12-year follow-up period extended into adolescence, and allowed for the longitudinal detection of persistent effects of maternal depression during a sensitive period in the pre-school years.
